# Glucosylceramide Synthase Is Involved in Development of Invariant Natural Killer T Cells

**DOI:** 10.3389/fimmu.2017.00848

**Published:** 2017-07-21

**Authors:** Zoran V. Popovic, Mariona Rabionet, Richard Jennemann, Damir Krunic, Roger Sandhoff, Hermann-Josef Gröne, Stefan Porubsky

**Affiliations:** ^1^Cellular and Molecular Pathology, German Cancer Research Center, Heidelberg, Germany; ^2^Institute of Pathology, University Medical Center Mannheim, University of Heidelberg, Mannheim, Germany; ^3^Light Microscopy Facility, German Cancer Research Center, Heidelberg, Germany

**Keywords:** CD1, glycosphingolipid, glucosylceramide, glucosylceramide synthase, natural killer T cell, thymus

## Abstract

Invariant natural killer T (iNKT) cells represent a unique population of CD1d-restricted T lymphocytes expressing an invariant T cell receptor encoded by Vα14-Jα18 and Vα24-Jα18 gene segments in mice and humans, respectively. Recognition of CD1d-loaded endogenous lipid antigen(s) on CD4/CD8-double positive (DP) thymocytes is essential for the development of iNKT cells. The lipid repertoire of DP thymocytes and the identity of the decisive endogenous lipid ligands have not yet been fully elucidated. Glycosphingolipids (GSL) were implicated to serve as endogenous ligands. However, further *in vivo* investigations were hampered by early embryonal lethality of mice deficient for the key GSL-synthesizing enzyme glucosylceramide (GlcCer) synthase [GlcCer synthase (GCS), EC 2.4.1.80]. We have now analyzed the GSL composition of DP thymocytes and shown that GlcCer represented the sole neutral GSL and the acidic fraction was composed of gangliosides. Furthermore, we report on a mouse model that by combination of Vav-promoter-driven iCre and floxed GCS alleles (*Vav^**Cre**^GCS^**f/f**^*) enabled an efficient depletion of GCS-derived GSL very early in the T cell development, reaching a reduction by 99.6% in DP thymocytes. Although the general T cell population remained unaffected by this depletion, iNKT cells were reduced by approximately 50% in thymus, spleen, and liver and showed a reduced proliferation and an increased apoptosis rate. The Vβ-chains repertoire and development of iNKT cells remained unaltered. The GSL-depletion neither interfered with expression of CD1d, SLAM, and Ly108 molecules nor impeded the antigen presentation on DP thymocytes. These results indicate that GlcCer-derived GSL, in particular GlcCer, contribute to the homeostatic development of iNKT cells.

## Introduction

Natural killer T (NKT) cells represent a unique T cell population co-expressing NK cell markers such as NK1.1 (CD161) (1, 2). Initially, a subset of NKT cells bearing an invariant T cell receptor (TCR) α-chain (Vα14-Jα18 in mouse and Vα24-Jα18 in human) paired with a limited repertoire of β-chains (Vβ2, Vβ7, Vβ8.2 in mouse and Vβ11 in human) could be identified, hence the designation as invariant NKT cells [invariant natural killer T (iNKT) or type I NKT] (3–6). iNKT cells are important mediators of tumor surveillance, peripheral tolerance and antimicrobial defense (7–15).

In contrast to conventional T cells, iNKT cells recognize lipid antigens presented by non-polymorphic MHC class I-like CD1 molecules (16, 17). Human genome encodes for five CD1 molecules that—based on the amino acid sequence—can be assigned to either group I (CD1a, -b, -c, and -e) or group II (CD1d) (18). Mice lack group I CD1 molecules and have two group II *Cd1* genes termed *Cd1d1* and *Cd1d2*, from which only *Cd1d1* seems to encode for a functional protein (19). Whereas presentation of peptide antigens on MHC molecules of thymic cortical epithelial cells is a prerequisite for the development of conventional T cells, positive selection of iNKT cells requires presentation of lipid antigens by CD1 molecules of double positive (CD4^+^/CD8^+^) thymocytes (20–22). In addition, lysosomal proteases and sphingolipid activator proteins, also known as saposins, are indispensable for normal thymic iNKT cell development suggesting that loading of lipid antigens onto CD1 molecules plays a crucial role in this process (23–26).

Several microbial, i.e., exogenous, lipid antigens recognized by iNKT cells have been identified (27, 28). α-Galactosylceramide (αGalCer, also referred to as KRN7000), which is derived from the marine sponge *Agelas mauritanius*, is the most potent member of this group (29, 30). Other α-anomeric microbial lipids with striking structural similarities to αGalCer and stimulatory effects toward iNKT cells have been found in *Sphingomonas* spp. (31, 32), *Borrelia burgdorferi* (33), and *Streptococcus pneumoniae* (34).

By contrast, lipid antigens mediating positive selection and peripheral homeostasis of iNKT cells are obviously of endogenous and not of microbial origin as implicated by the fact that germ-free mice show an unaltered iNKT cell population (35). A variety of endogenous lipids (mostly phospholipids and sphingolipids) have been shown to be captured by CD1d during endosomal–lysosomal recycling or on the secretory pathway (36–39). However, most iNKT cells do not respond to these lipids and the reactivity toward them is restricted to singular iNKT cell clones (40).

**Figure 1:**
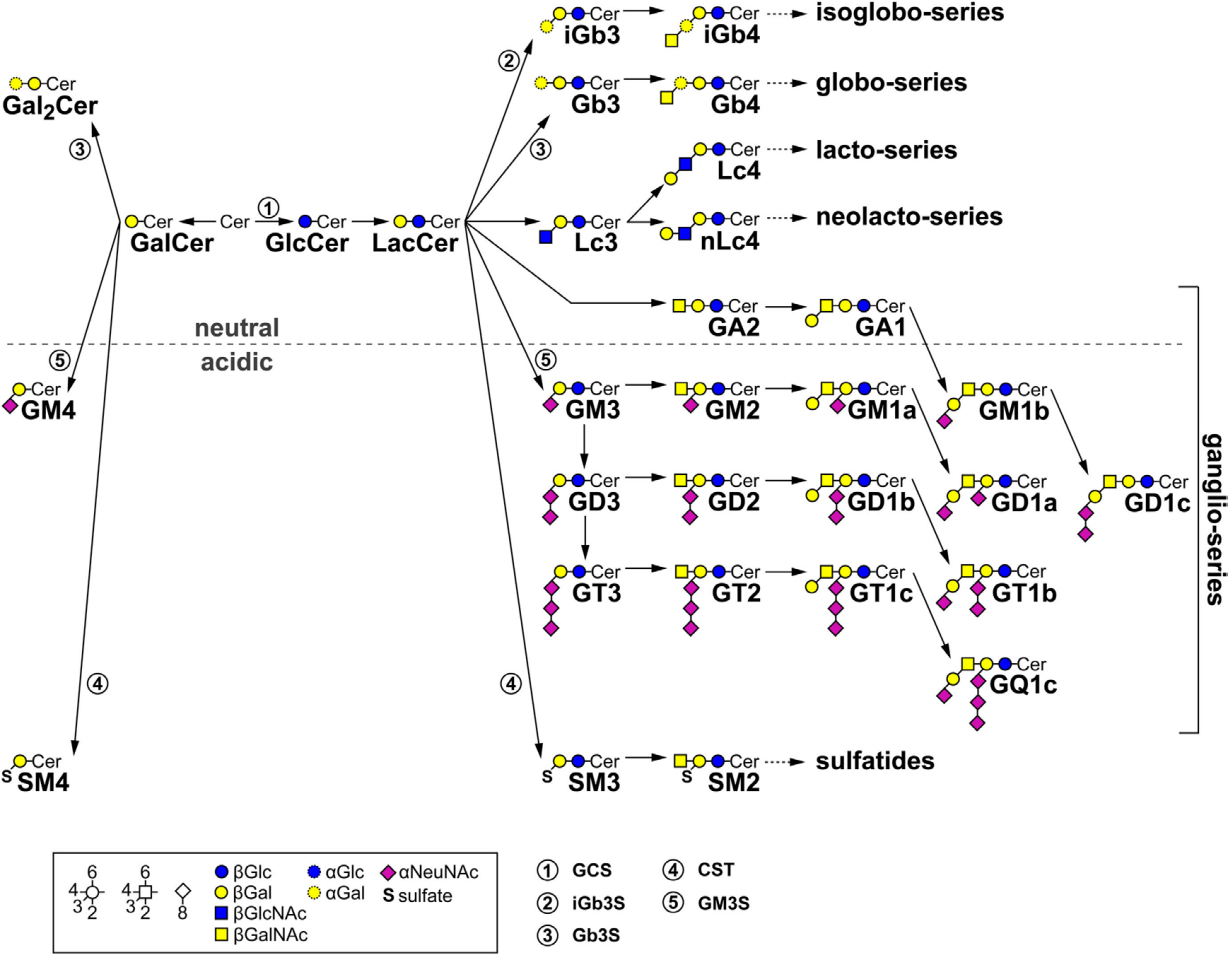
Metabolic glycosphingolipid (GSL) pathways. The diagram shows the most important mammalian metabolic GSL pathways starting from ceramide (Cer). Depending on the first sugar moiety, either galactosylceramide (GalCer) or glucosylceramide (GlcCer) are formed. GlcCer is processed to lactosylceramide (LacCer). By subsequent action of further enzymes on either GalCer or LacCer, individual series of GSL emerge. The presence of an acidic moiety [*N*-acetylneuraminic acid (αNeuNAc) in gangliosides or sulfate (S) in sulfatides, respectively] results in an acidic character of these GSL. Relevant synthesis enzymes are indicated as encircled numbers: 1, GlcCer synthase (GCS); 2, isoglobotriaosylceramide (iGb3) synthase (iGb3S); 3, globotriaosylceramide (Gb3) synthase (Gb3S); 4, cerebroside sulfotransferase (CST); and 5, GM3 synthase (GM3S).

Despite an extensive research, the identity of the endogenous lipid antigen(s) responsible for the thymic selection of iNKT cells remains partially unresolved (41, 42). It has been demonstrated that mice deficient for glyceronephosphate O-acyltransferase (GNPAT) show an altered iNKT cell development (43). Based on the observation that cells deficient in glucosylceramide (GlcCer)-based glycosphingolipids (GSL) (Figure 1) were unable to stimulate iNKT cell hybridomas, it was suggested that the endogenous selecting ligand might be GlcCer or a GlcCer-derived GSL (44). Subsequent studies pinpointed to GlcCer as an endogenous lipid antigen mediating activation of iNKT cells in response to microbial danger signals (45). However, later, the same group reported that a minor—hitherto unidentified—lipid co-purifying with GlcCer might function as the actual self-lipid antigen (46). Until now, *in vivo* experiments addressing the putative role of GlcCer-derived GSL during thymic iNKT cell development were hampered by an early embryonic lethality of mice deficient for GlcCer synthase (GCS) (47).

In the present study, we have analyzed the GSL composition of double-positive (DP) thymocytes and shown that besides GlcCer, these cells expressed GlcCer-derived acidic GSL from the ganglio series such as GM1a, GM1b, GD1b, and GD1c. Furthermore, we have circumvented the lethality of GCS-deficient embryos by investigating mice with tissue-specific deletion of the GCS gene (*Vav^Cre^GCS^f/f^*) and demonstrated that depletion of GlcCer-derived GSL in DP thymocytes resulted in a significant reduction of the iNKT cell population. Thus, GlcCer-derived GSL represent relevant endogenous lipids contributing to the development of iNKT cells.

## Materials and Methods

### Experimental Mice

Mice with floxed GCS (Ugcg, EC 2.4.1.80) alleles were described previously (48). TCRVα14-Jα281 transgenic mice were kindly provided by Agnes Lehuen (49). CD1d-deficient (50) and *Vav^Cre^*-transgenic (51) mice were purchased from The Jackson Laboratory (Bar Harbor, ME, USA). All strains were backcrossed for more than 10 generations to the C57BL/6 genetic background (Charles River Wiga, Sulzfeld, Germany) and housed under specific pathogen-free conditions. *Vav^Cre^*-negative littermates were used as wild-type (WT) controls. Animal experiments were performed in compliance with the German guidelines on animal protection.

### Organ Preparation, Flow Cytometry, and Cell Sorting

Single cell preparations from organs were prepared as described previously (52). Flow cytometry was performed as described in Ref. (53). The following monoclonal antibodies were used: anti-CD1d (clone: 1B1); anti-CD3ε (145-2C11), anti-CD4 (GK1.5), anti-CD8 (53-6.7), anti-CD11c (HL3), anti-CD19 (MB19-1), anti-CD25 (PC61.5), anti-CD44 (IM7), anti-CD150/SLAM (9D1), anti-Ly108 (13G3), anti-MHCII (M5/144.15.2), anti-NK1.1 (PK136), anti-TCR-Vβ2 (B20.6), anti-TCR-Vβ7 (TR310), and anti-TCR-Vβ8.1 and 8.2 (MR5-2) from BD, Heidelberg, Germany, Biolegend, San Diego, CA, USA, and eBioscience, San Diego, CA, USA. PBS57-loaded PE-labeled CD1d tetramers were kindly provided by NIH Tetramer Core Facility at Emory University (Atlanta, GA, USA). BrdU and Annexin V experiments were performed according to the manufacturer’s protocol (both BD). Analysis of flow cytometry data was performed using Cell Quest Pro software (BD) and FlowJo (Tree Star, Flow Cytometry Analysis Software) by gating on lymphocytes in the forward and side scatter. Double-positive thymocytes were sorted using FACSAria™ (BD) by gating stringently on CD4^+^/CD8^+^ DP lymphocytes and excluding 7AAD-positive dead cells.

### RNA Isolation and Quantitative PCR

RNA was extracted from cell pellets using the phenol/chloroform extraction method (54) followed by digestion by RNase-free DNaseI (turbo DNA free, Ambion, Huntingdon, UK). A total of 3 µg of total RNA were reverse transcribed in 20-µl total volume using SuperscriptII (Invitrogen, Karlsruhe, Germany) according to the manufacturers’ instructions. RT-PCR was performed with 1 µl cDNA and GCS primers: forward 5′—gat cta aga ggg tga agg cgc a—3′ and reverse 5′—ctg cct tgc aat cct gtc tgt c—3′.

### Isolation and Analysis of GSL

Glycosphingolipids were extracted from lyophilized cell pellets as described in detail in Ref. (55, 56). For thin layer chromatography (TLC) analysis, an amount corresponding to 0.2 mg protein was loaded on a TLC plate (Merck, Darmstadt, Germany). Running solvent was CHCl_3_/CH_3_OH/H_2_O (62.5:30:6, v/v/v) for neutral GSL and CHCl_3_/CH_3_OH/0.2% CaCl_2_ in H_2_O (60:35:8, v/v/v) for acidic GSL, respectively. Sialidase treatment was performed as described in Ref. (57). 0.05 U *Vibrio cholerae* sialidase in 0.2 M Na-acetate buffer, 2 mM CaCl_2_, pH 5.2, was used to digest acidic GSL on a polyisobutylmethacrylate-fixed TLC plate at room temperature for 8 h.

### Mass Spectrometric Analyses

Sphingolipids from DP thymocytes were extracted as previously described with slight modifications (58). Briefly, sorted thymocytes (~5 × 10^6^) were dried with 1-propanol and extracted twice at 37°C for 15 min with a chloroform/methanol/water mixture of 10/10/1 (v/v/v) and once with 30/60/8. The residual cell pellets were used for protein determination according to the Lowry method. The combined lipid extracts were dried under air flow and subsequently subjected to mild alkaline hydrolysis with 0.1 M potassium hydroxide in methanol for 2 h at 37°C. Saponified extracts were finally desalted by reverse-phase (C18) column chromatography. Aliquots corresponding to 30 µg of protein were dissolved in 1 ml 95% methanol containing the following internal standard mixture: Cer (d18:1;14:0), Cer (d18:1;19:0), Cer (d18:1;25:0), Cer (d18:1;31:0) each 4 pmol; GlcCer (d18:1;14:0), GlcCer (d18:1;19:0), GlcCer (d18:1;25:0), and GlcCer (d18:1;31:0) each 2 pmol.

For quantification of lipid extracts, UPLC–ESI–MS/MS analyses were performed as described in Ref. (59) with following modifications: lipid extracts were separated in a reverse-phase (C18) column, which was kept at 45°C, while the autosampler was maintained at 15°C. After equilibration with buffer A (95% methanol, 0.05% formic acid, and 1 mM ammonium formate), lipids were eluted with increasing percent of buffer B (99% 2-propanol, 1% methanol, 0.05% formic acid, and 1 mM ammonium formate) up to 85%. Ceramide and hexosylceramide species were detected with a precursor ion scan of *m*/*z* +264 corresponding to sphingosine (d18:1) while keeping the cone voltage at 50 V and the collision energy at 44 eV.

### *In Vitro* and *In Vivo* Experiments with iNKT and T Cells

Double-positive thymocytes were isolated from WT, *Vav^Cre^GCS^f/f^*, and *CD1d^–/–^* thymi after depleting cells reactive with PBS57-loaded CD1d tetramers. 0.5 × 10^6^ DP thymocytes per well were placed in 96-well-plate and incubated with αGalCer (Avanti Polar Lipids, Alabaster, AL, USA) at indicated concentrations. iNKT cells were enriched from livers of TCRVα14-Jα281 transgenic mice using anti-CD5 micro beads (Miltenyi Biotec) and applied at 50,000/well. Activation of T cells *in vitro* was performed as described in Ref. (60). Briefly, splenic T-cells were enriched by anti-CD90.2 micro beads (Miltenyi Biotec) and incubated with 0.5 mg/ml calcium ionophore A23187 and 10 ng/mL phorbol 12-myristate 13-acetate (PMA, both Sigma). Supernatants were collected after 18 h and analyzed for IFNγ and IL4 concentrations by cytometric bead array technique (BD). For the *in vivo* testing of iNKT cells function, mice were injected i.p. with 0.2 or 3 µg αGalCer and sacrificed 8 h later.

### Super-Resolution Microscopy

Thymocytes of WT and *Vav^Cre^GCS^f/f^* mice were enriched by magnetic separation using CD5 beads (Miltenyi), spinned down using cytospin system (4 × 10^5^ cells/slide) and fixed in 1% paraformaldehyde in PBS for 15 min at room temperature. Cells were then incubated with antibodies against CD1d-FITC (BD), early endosome antigen 1 (EEA1) (Cell Signaling), Rab7 (Santa Cruz Biotechnology), and lysosome-associated membrane protein 1 (LAMP1) (eBioscience). After washing, corresponding Alexa-Fluor 546-conjugated secondary antibodies were added and the slides were incubated for 1 h at room temperature in the dark. DAPI was used for nuclear visualization. Negative controls contained DAPI staining and Alexa-Fluor 546-conjugated secondary antibodies (for EEA1, Rab7, and LAMP1), or DAPI staining only (for CD1d). Images were acquired using the Olympus IX81 motorized microscope equipped with the MT20 illumination system; the Cy3, GFP, and DAPI HC-Filter sets; and Hamamatsu Orca-ER CCD camera. Two hundred images from each channel were acquired for each region using the 100×/1.4 PlanApo objective, and five regions were analyzed from each sample. Images were post-processed with ImageJ (http://rsbweb.nih.gov/ij) to obtain super-resolution optical fluctuation images—www.ncbi.nlm.nih.gov/pubmed/20018714. On average, 20 cells were analyzed for co-localization between red and green using the ImageJ’s co-localization plugin, and the ratio of co-localized and total green area was plotted and statistically analyzed. The images of lysosomes were further analyzed automatically with the same parameters using ImageJ macro developed at DKFZ Light Microscopy Core Facility (Heidelberg, Germany). Shortly, images of lysosomes were thresholded and segmented using the Find Maxima tool with the Segmented Particles above lower threshold option activated. The segmented particles above the minimum area limit of 10 pixels (0.022 µm^2^) were further counted for each cell using ImageJ’s Analyze Particles tool.

### Statistical Analysis

Unpaired two-tailed Student’s *t*-test was performed to compare data sets. Differences were considered significant if *p* < 0.05. Numbers of independent observations per group are indicated for each result.

## Results

### GSL Composition of DP Thymocytes

**Figure 2:**
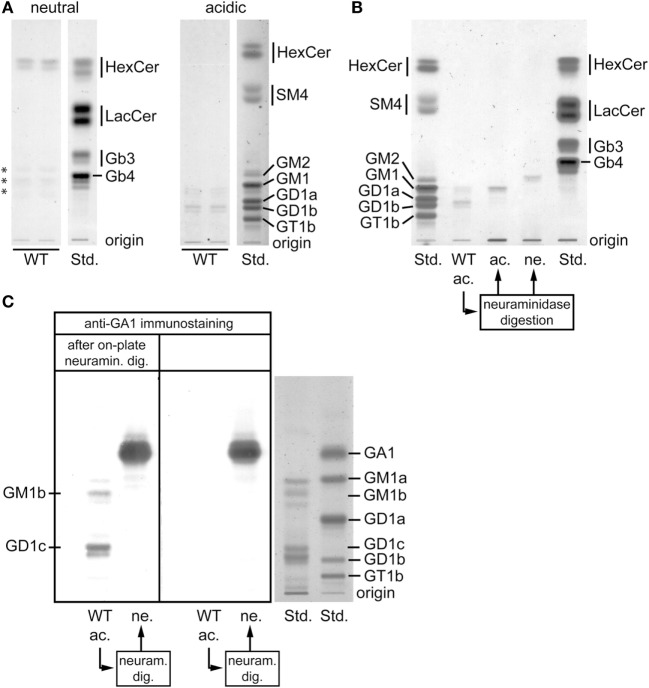
Glycosphingolipid (GSL) composition of double-positive (DP) thymocytes. **(A)** Neutral and acidic GSL were extracted from enriched wild-type (WT) DP thymocytes and analyzed by thin layer chromatography (TLC). Some GSL are represented by multiple bands due to their heterogeneous composition of ceramide moieties resulting in different running properties. The substances in the neutral fraction marked by asterisks did not show the typical orcinol color for GSL and therefore very unlikely represent GSL compounds. The displayed lanes represent parts of the chromatograms that are shown and further analyzed in Figure 3A. Orcinol staining. **(B)** The acidic GSL from WT DP thymocytes were digested by neuraminidase, separated into an acidic (ac.) and a neutral (ne.) fraction and ran in parallel with the original acidic fraction from WT DP thymocytes (WT ac.). The acidic compound obtained after the neuraminidase digestion ran at the height of the GM1 standard. By contrast, the neutral compound was not represented in the standards; however due to its running properties, it corresponded likely to neutral ganglioside GA1. Orcinol staining. **(C)** To verify from which acidic GSL of WT DP thymocytes its neutral backbone GA1 was released, an immunostaining with anti-GA1 antibodies was performed after additional “on-plate” neuraminidase digestion (left panel). Both the upper and the lower band contained GA1-based compounds. In view of this fact and the running properties, these bands likely correspond to GM1a, GM1b, GD1b, and GD1c. The part of the TLC plate containing GSL standards (Std.) was stained by orcinol.

Because the development of iNKT cells depends on presentation of lipid antigens on DP thymocytes, we analyzed the latter cell population for its GSL composition in WT mice. In the neutral GSL fraction, hexosylceramides represented the major fraction (Figure 2A). In the acidic fraction, two compounds running at the height of GM1 and GD1, respectively, emerged (Figure 2A). In order to further characterize these substances, the acidic fraction was digested by neuraminidase and the products were subsequently separated into acidic and neutral fractions (Figure 2B). The hereby obtained acidic compound ran at the height of the GM1 standard suggesting that this band probably consisted of a mixture of non-digestible GM1 or was derived from GD1b after the release of the terminal sialic acid. By contrast, the neutral compound was not represented in the standards; however, due to its running properties, it likely corresponded to neutral ganglioside GA1 (Figure 2B). To identify this product, the original acidic fraction was on-plate digested with neuraminidase and subsequently immunostained using anti-GA1 antibodies (Figure 2C). Both the upper and the lower compounds had a neutral backbone of GA1 (Figure 2C, left panel). Based on the running properties and the comparison with the standards, these compounds likely correspond to GM1a, GM1b, GD1b, and GD1c.

### Characterization of *Vav^Cre^GCS^f/f^* Mice

Although several lines of evidence have implicated that GlcCer-based GSL might belong to the lipid antigens relevant for the iNKT cell development, a direct proof of this hypothesis was precluded by the early embryonal lethality of GCS-deficient mice (47). To overcome this problem, we implemented a tissue-specific deletion of this gene. To this end, mice with floxed alleles of the GCS gene (*GCS^f/f^*) were crossed with mice expressing iCre under the control of the Vav-promoter (*VavCre*) that activates the recombinase activity very early in T-cell development with virtually 100% of DN1 (CD25^−^/CD44^+^) thymocytes being already positive (51).

**Figure 3:**
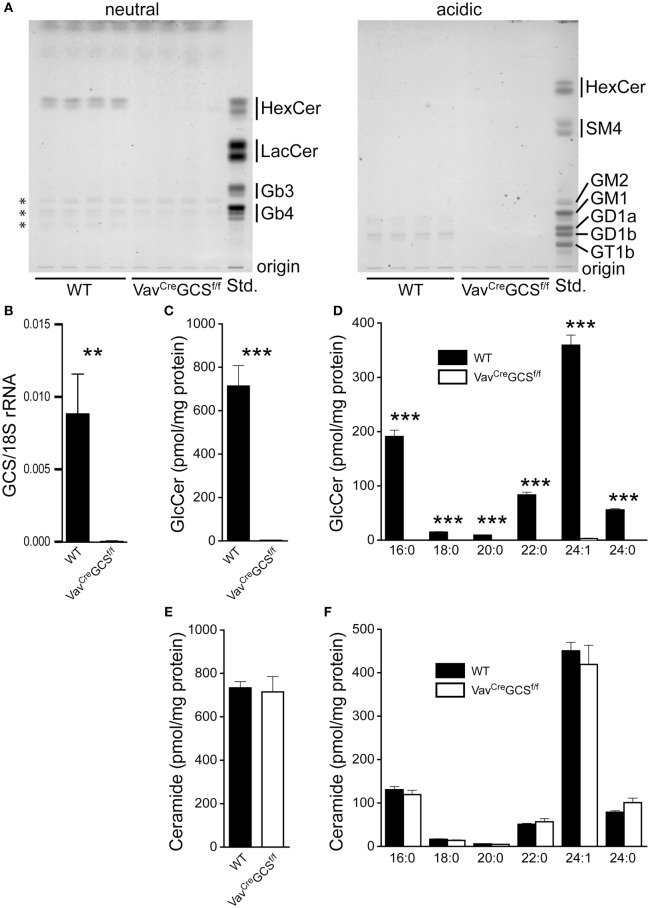
Glycosphingolipid (GSL) depletion in *Vav^Cre^GCS^f/f^* double-positive (DP) thymocytes. **(A)** GSL were extracted from enriched DP thymocytes from 8-week-old wild-type (WT) and *Vav^Cre^GCS^f/f^* mice, separated into neutral and acidic fractions and analyzed by thin layer chromatography. In DP thymocytes from *Vav^Cre^GCS^f/f^* mice, no residual GSL could be detected. The substances in the neutral fraction marked by asterisks do not show the typical orcinol color for GSL and therefore very unlikely represent those compounds. Shown are results from four different experimental animals per group. **(B)** FACS-sorted DP thymocytes were analyzed for the expression of GlcCer synthase (GCS) by quantitative PCR. The expression was normalized to 18S rRNA. Shown are means ± SEM, *N* = 7 per group. **(C–F)** GSL were extracted from FACS-sorted DP thymocytes. The content of GlcCer **(C,D)** and ceramide **(E,F)** was quantified by mass spectrometry and normalized for the protein amount in the sample. Panels **(C,E)** show the total amount of GlcCer and ceramide, respectively. In panels **(D,F)**, the composition of acyl moieties of GlcCer and ceramide, respectively, are displayed. Some of the bars for *Vav^Cre^GCS^f/f^* are barely visible due to very low levels. Shown are means ± SEM, *N* = 6 and 7 per group, respectively. Statistically significant differences between WT and *Vav^Cre^ GCS^f/f^* mice are indicated: **p < 0.01; ***p < 0.001.

In terms of TLC analysis, no GSL could be detected in DP thymocytes from *Vav^Cre^GCS^f/f^* mice (Figure 3A). This was in line with extensive and significant reduction of GCS mRNA in these cells (Figure 3B). Mass spectrometry performed on FACS-sorted DP thymocytes revealed a 99.6% reduction of the GlcCer content in *Vav^Cre^GCS^f/f^* mice as compared to WT (Figure 3C). This reduction occurred independently of the analyzed acyl moiety (Figure 3D). By contrast, the ceramide content of *Vav^Cre^GCS^f/f^* DP thymocytes was indistinguishable from WT (Figures 3E,F).

**Figure 4:**
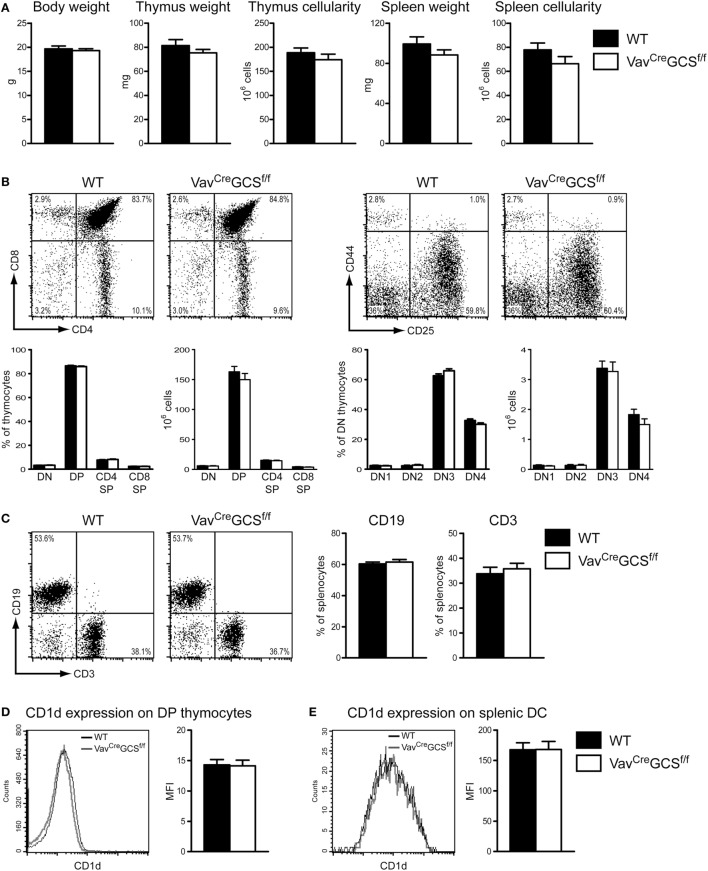

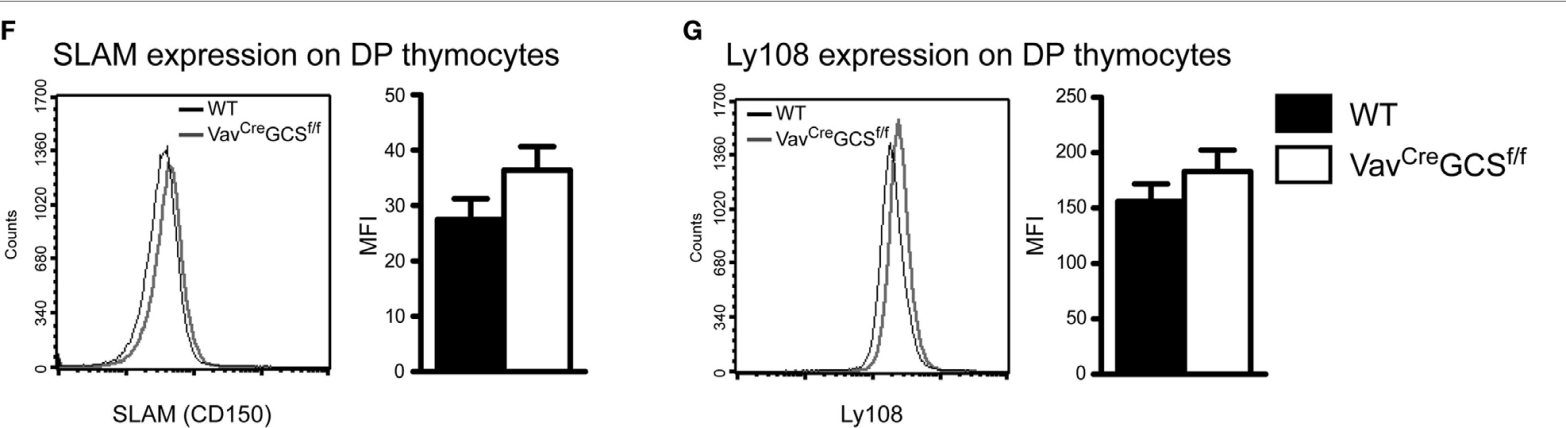
*Vav^Cre^GCS^f/f^* mice showed a typical thymocyte development and an unaltered expression of CD1d, SLAM and Ly108 molecules. **(A)** No statistically significant differences were observed between 8 week old WT and *Vav^Cre^GCS^f/f^* mice in terms of body weight and the weight and cellularity of spleen and thymus. Bars show means ± SEM, *N* = 6 per group. **(B)** Thymocyte development was investigated in 8 week old mice by flow cytometry using antibodies against CD4 and CD8. The double-negative (DN) stage was further subdivided into DN1 (CD25^–^/CD44^+^), DN2 (CD25^+^/CD44^+^), DN3 (CD25^+^/CD44^–^), and DN4 (CD25^–^/CD44^–^). Representative dot plots (upper panels) as well as relative and absolute numbers (lower panels) are shown (mean ± SEM, *N* = 13 per group). No statistically significant differences could be observed between WT and *Vav^Cre^GCS^f/f^* mice. **(C)** Frequencies of B (CD3^–^/CD19^+^) and T (CD3^+^/CD19^–^) cells were measured by flow cytometry in spleens of 8 week old mice. Representative dot-plots and quantifications are shown (mean ± SEM, *N* = 13 per group). No statistically significant differences could be observed between WT and *Vav^Cre^GCS^f/f^* mice. **(D,E)** Expression of CD1d was measured on DP (CD4^+^/CD8^+^) thymocytes **(D)** and splenic DC (CD11c^+^/MHCII^+^) **(E)** and expressed as mean fluorescence intensity (MFI). No statistically significant difference could be observed between *WT* and *Vav^Cre^GCS^f/f^* mice. Shown are means ± SEM, *N* = 6 per group in panel **(D)** and 4 per group in panel **(E)**. **(F,G)** Expression of SLAM (CD150) and Ly108, respectively, was measured on DP (CD4^+^/CD8^+^) thymocytes and expressed as MFI. No statistically significant difference could be observed between *WT* and *Vav^Cre^GCS^f/f^* mice. Shown are means ± SEM, *N* = 4 per group.

*Vav^Cre^GCS^f/f^* mice reproduced normally and progeny were born at expected Mendelian ratios (data not shown). Newborn and adult *Vav^Cre^GCS^f/f^* mice did not exhibit any overt growth, developmental or behavioral defects. Body weight, the weight, and cellularity of thymus and spleen were indistinguishable from *Vav^Cre^*-negative littermates (Figure 4A). Similarly, no aberration in the maturation of the conventional thymocytes could be revealed by flow cytometry in *Vav^Cre^GCS^f/f^* mice (Figure 4B). In spleens, the amount of CD3- and CD19-positive T- and B-lymphocytes, respectively, was unaffected by the deletion of the GCS gene (Figure 4C). The expression levels of CD1d on *Vav^Cre^GCS^f/f^* DP thymocytes and splenic CD11c^+^/MHCII^+^ dendritic cells were indistinguishable from WT mice (Figures 4D,E). The expression of SLAM (CD150) and Ly108 molecules, which provide important signals on DP thymocytes during the thymic iNKT cell development (61), did not significantly differ between *Vav^Cre^GCS^f/f^* and WT mice (Figures 4F,G).

**Figure 5:**
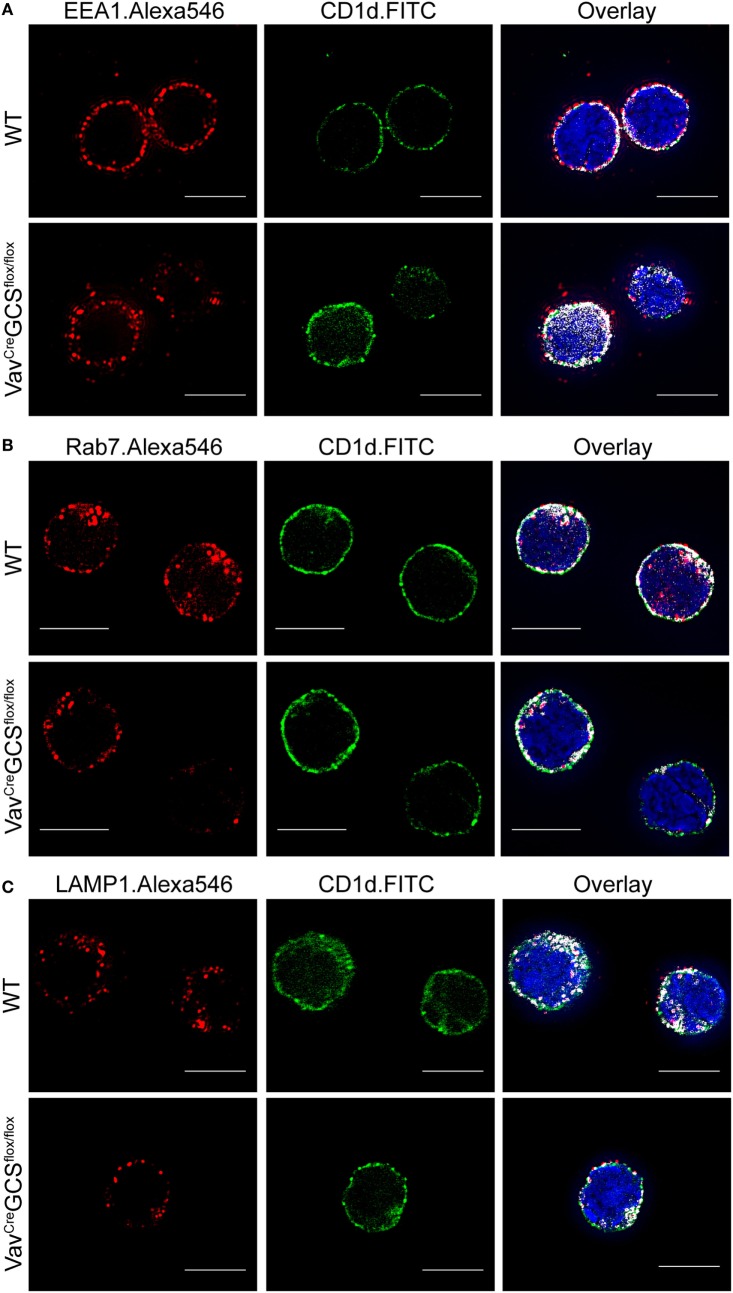

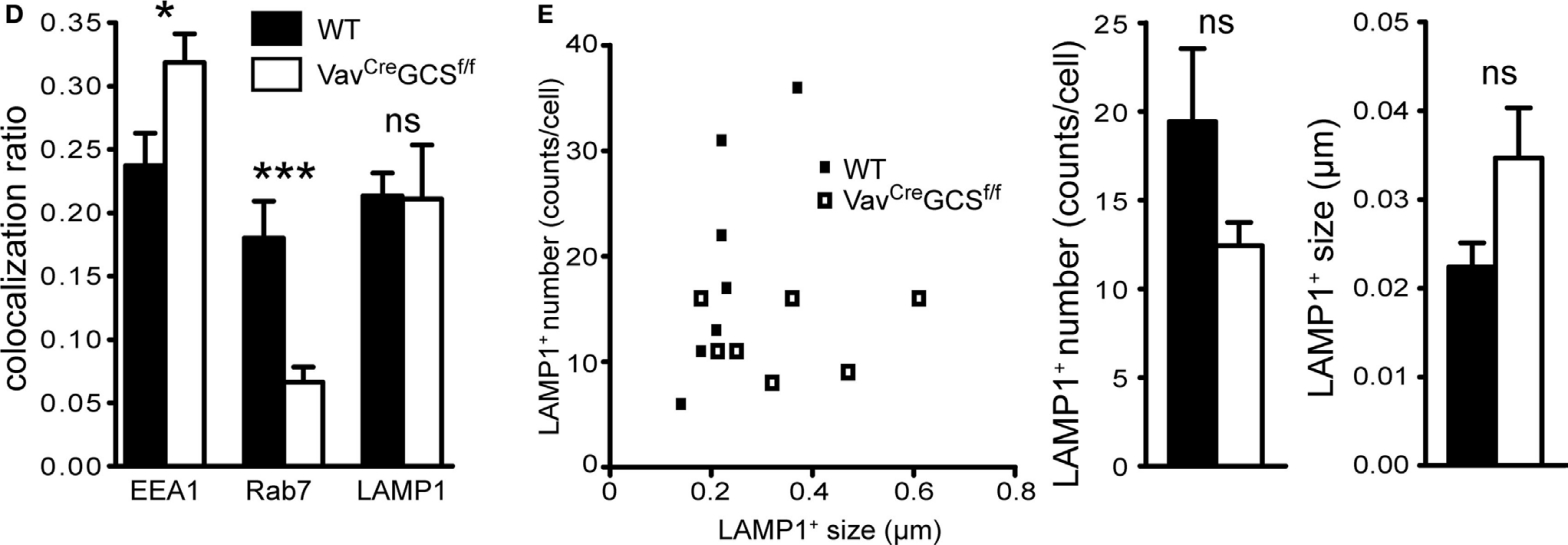
CD1d trafficking through early endosomes, late endosomes, and lysosomes. **(A–C)** In double-positive (DP) thymocytes, super-resolution microscopy was used to determine intracellular localization of CD1d molecules in early endosomes, late endosomes, and lysosomes visualized in the red channel by early endosome antigen 1 (EEA1), Rab7, and lysosome-associated membrane protein 1 (LAMP1), respectively. Co-localization areas were presented in white (right panels). DAPI was applied to visualize the nucleus (bar = 5 µm). **(D)** DP thymocytes were analyzed for co-localization between green and red signals using the ImageJ’s co-localization plugin, and the ratio of co-localized and total green area was plotted and statistically analyzed using the unpaired t-test. Although a significant shift from late to toward early endosomes could be observed in *Vav^Cre^GCS^f/f^* DP thymocytes, the amount of CD1d in lysosomes was equal. Shown are means ± SEM, *N* = 20 cells per group. **(E)** A tendency toward less but larger LAMP1^+^ lysosomes could be seen in DP thymocytes of *Vav^Cre^GCS^f/f^* mice, however, the difference was not statistically significant. The bars show means ± SEM, *N* = 7 cells per group.

Furthermore, CD1d trafficking was analyzed using super-resolution microscopy and EEA1, Rab7, and LAMP1 as markers of early endosomes, late endosomes and lysosomes, respectively (Figures 5A–C). The quantification of signal co-localization revealed a statistically significant shift of CD1d from late to early endosomes in *Vav^Cre^GCS^f/f^* mice. In contrast, the CD1d amount in lysosomes remained unaffected (Figure 5D). Although a tendency toward less but larger lysosomes could be seen in DP thymocytes of *Vav^Cre^GCS^f/f^* mice, the difference was not statistically significant (Figure 5E).

### Significant Reduction of the iNKT Cell Population in *Vav^Cre^GCS^f/f^* Mice

**Figure 6:**
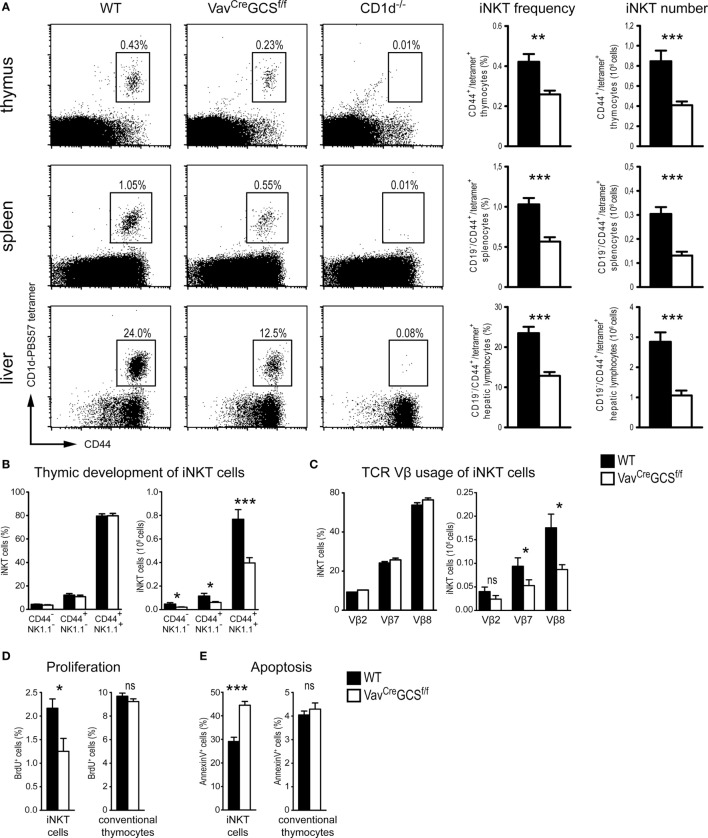
*Vav^Cre^GCS^f/f^* showed a significant reduction of the invariant natural killer T (iNKT) cell population. **(A)** In thymi, spleens, and livers of 8-week-old wild-type (WT), *Vav^Cre^GCS^f/f^* and *CD1d^–/–^* mice, frequencies and absolute numbers of iNKT cells were measured by flow cytometry using PBS57-loaded CD1d tetramers and anti-CD44 antibodies. In spleens and livers, CD19^+^ cells were gated out. In *Vav^Cre^GCS^f/f^* mice, iNKT cells frequencies and numbers were significantly reduced in all three organs. CD1d-deficient mice served as negative controls. *N* = 10–13/group. **(B)** Thymic development of iNKT cells was investigated in 8-week-old mice. Antibodies against NK1.1 and CD44 were used to subdivide the developmental stages in immature (CD44^−^/NK1.1^−^), semi-mature (CD44^+^/NK1.1^−^), and mature (CD44^+^/NK1.1^+^). Analyses were gated on iNKT cells defined as CD3^+^/PBS57-CD1d^+^ thymocytes. Shown are relative and absolute numbers (left and right panels, respectively) of iNKT cells with the corresponding phenotype. No statistically significant differences could be observed between WT and *Vav^Cre^GCS^f/f^* mice in terms of relative numbers (i.e., distribution among the three stages). The significant reduction in absolute numbers reflected the overall diminished iNKT cell population in *Vav^Cre^GCS^f/f^* mice. *N* = 16/group in the left panel and 10/group in the right panel, respectively. **(C)** Usage of TCRVβ-chains by splenic iNKT cells was investigated in 8-week-old mice. Analyses were gated on CD19^−^/PBS57-CD1d^+^/CD44^+^ splenocytes. Shown are relative and absolute numbers (left and right panels, respectively) of iNKT cells expressing the corresponding TCRVβ-chain. No statistically significant differences could be observed between WT and *Vav^Cre^GCS^f/f^* mice in terms of relative numbers (i.e., distribution among the three TCRVβ-chains). The reduction in the absolute numbers reflected the diminished iNKT cell population in *Vav^Cre^GCS^f/f^* mice. *N* = 9/group in the left panel and 6/group in the right panel, respectively. **(D,E)** Proliferation and apoptosis of thymic iNKT cells were measured in 8-week-old mice using BrdU incorporation and Annexin V staining, respectively. In *Vav^Cre^GCS^f/f^* mice, iNKT cells (CD3^+^/PBS57-CD1d^+^ thymocytes) showed a significantly reduced proliferation and an increased apoptosis as compared to WT controls. By contrast, conventional thymocytes were unaffected. *N* = 5/group. Bars represent means ± SEM; *p < 0.05; **p < 0.01; ***p < 0.001; ns, non-significant.

In newborn and adult mice, the iNKT cell populations were characterized by flow cytometry using PBS57-loaded CD1d tetramers. Adult *Vav^Cre^GCS^f/f^* mice showed a significant reduction of the iNKT population in terms of absolute numbers and percentages in thymus, spleen, and liver as compared to WT littermates (Figure 6A). In all three organs, a reduction of the iNKT cell population by approximately 50% could be observed. In *Vav^Cre^GCS^f/f^* mice, the remaining iNKT cells could be clearly identified and discerned from any unspecific staining as visualized by comparison with CD1d-deficient mice that do not produce iNKT cells (Figure 6A). Newborn mice showed identical results (data not shown). To test for possible unspecific effects of the *Vav^Cre^* transgene, iNKT cell frequencies and absolute numbers were compared between *Vav^Cre^*-positive and *Vav^Cre^*-negative *GCS^+/+^* mice showing no statistically significant differences (Table S1 in Supplementary Material).

In course of thymic maturation, iNKT cells upregulate expression of NK1.1 and CD44 allowing the identification of three developmental stages: immature, CD44^−^/NK1.1^−^; semi-mature, CD44^+^/NK1.1^−^; and mature, CD44^+^/NK1.1^+^ (62). In terms of absolute numbers, iNKT cells were significantly reduced in all three developmental stages in *Vav^Cre^GCS^f/f^mice*. However, no significant difference was observed in the percentual distribution among these three stages (Figure 6B).

In iNKT cells, the invariant Vα14-chain pairs almost exclusively with Vβ2, 7, or 8.2 (63). We tested whether the depletion of GCS-derived GSL would lead to a shift of the Vβ-chain repertoire in *Vav^Cre^GCS^f/f^* mice. However, no statistically significant difference in the percentage distribution of the Vβ-chains could be found between *Vav^Cre^GCS^f/f^* mice and WT littermates. In terms of absolute numbers, a decrease corresponding to the diminished iNKT cell population could be observed (Figure 6C).

Measurements of proliferation and apoptosis rate by BrdU incorporation and Annexin V staining, respectively, revealed that in *Vav^Cre^GCS^f/f^* mice, thymic iNKT cells showed a significantly reduced proliferation and an increased apoptosis as compared to WT mice. By contrast, conventional thymocytes were unaffected (Figures 6D,E).

### Deletion of GCS Did Not Affect the Processes of Antigen Presentation and Recognition

**Figure 7:**
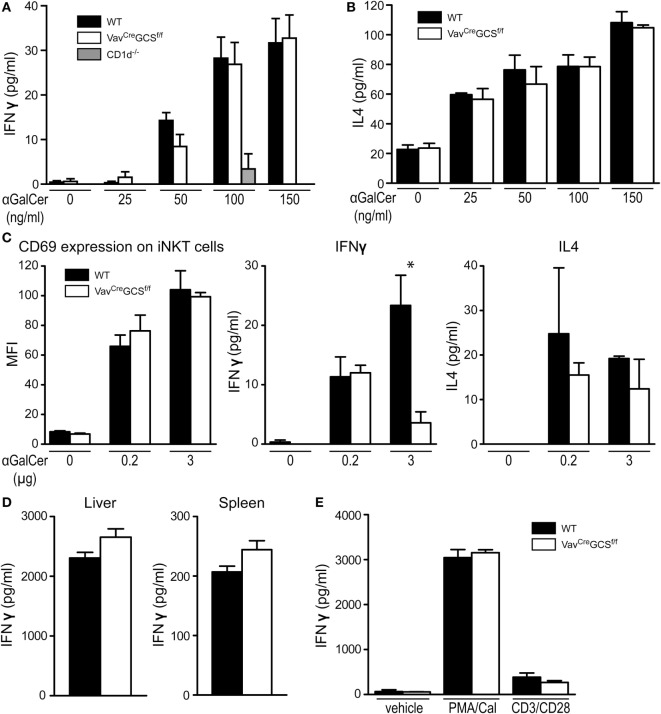
Antigen presentation and recognition in *Vav^Cre^GCS^f/f^* mice. **(A,B)** Double-positive (DP) thymocytes were tested for their antigen presentation capacity toward invariant natural killer T (iNKT) cells *in vitro*. To this end, iNKT-depleted wild-type (WT), *Vav^Cre^GCS^f/f^*, and *CD1d^–/–^* DP thymocytes were exposed to increasing concentrations of αGalCer and co-incubated with responder WT iNKT cells enriched from livers of TCRVα14-Jα281 transgenic mice. The activation measured as secretion of IFNγ **(A)** and IL4 **(B)** did not differ between WT and *Vav^Cre^GCS^f/f^* DP thymocytes. *CD1d^–/–^* DP thymocytes served as negative controls, and the corresponding bars cannot be discriminated from the zero line in all but one concentration. Shown are means ± SEM, *N* = 6–9 per group. **(C)** Activation of iNKT cells was tested *in vivo*. WT and *Vav^Cre^GCS^f/f^* mice were i.p. injected with either 0.2 or 3 µg αGalCer. Eight hours later, splenic iNKT cells were analyzed for surface CD69 expression by flow cytometry by gating on CD19^−^/PBS57-CD1d^+^/CD44^+^ lymphocytes. Expression of CD69 did not differ between WT and *Vav^Cre^GCS^f/f^* DP thymocytes. In parallel, serum was analyzed for IFNγ and IL4 levels. In *Vav^Cre^GCS^f/f^* mice injected with 3 µg αGalCer, IFNγ levels were significantly lower than in the WT controls. All other measurements did not show a statistically significant difference. Shown are means ± SEM, *N* = 3 per group. **(D)** Activation of iNKT cells was tested *in vitro*. iNKT cells from livers and spleens of WT and *Vav^Cre^GCS^f/f^* mice were exposed to αGalCer-loaded WT DP thymocytes. The activation measured as IFNγ secretion did not differ between WT and *Vav^Cre^GCS^f/f^* iNKT cells. Shown are means ± SEM, *n* = 3–6 per group. **(E)** Splenic conventional T cells were tested for their T cell receptor (TCR)-independent and TCR-dependent activation *in vitro*. WT and *Vav^Cre^GCS^f/f^* splenic T cells were activated by PMA/calcium ionophore A23187 or by plate-bound anti-CD3/anti-CD28 antibodies. Vehicle (media)-treated cells served as controls. No statistically significant differences could be found in the IFNγ secretion between WT and *Vav^Cre^GCS^f/f^* T cells. Shown are means ± SEM, *n* = 6 per group.

Depletion of GCS-derived GSL in DP thymocytes might not only alter the repertoire of lipid antigens but also impact the processes of their presentation. Thus, in order to test the antigen presenting capacity of *Vav^Cre^GCS^f/f^* DP thymocytes, these cells were exposed to increasing concentrations of the exogenous antigen αGalCer and co-incubated with WT responder iNKT cells enriched from livers of TCRVα14-Jα281 transgenic mice. As measured by secretion of IFNγ and IL4, no statistically significant difference could be observed between the antigen presentation on DP thymocytes from *Vav^Cre^GCS^f/f^* and WT mice (Figures 7A,B).

Furthermore, we have subjected *Vav^Cre^GCS^f/f^* iNKT cells to functional tests *in vivo* and *in vitro*. Upon injection of αGalCer, upregulation of CD69 on iNKT cells was unaltered in *Vav^Cre^GCS^f/f^* mice (Figure 7C). IFNγ levels were significantly lower in *Vav^Cre^GCS^f/f^* mice injected with 3 µg αGalCer. Similarly, IL4 levels tended to be lower in the *Vav^Cre^GCS^f/f^* mice although a statistical significance was not reached (Figure 7C). Therefore, we tested the reactivity of *Vav^Cre^GCS^f/f^* iNKT cells *in vitro* with equalized cell numbers. To this end, WT DP thymocytes were loaded with αGalCer and co-incubated with iNKT cells enriched from spleens and livers of *Vav^Cre^GCS^f/f^* or WT mice. No functional deficiency could be observed between iNKT cells from *Vav^Cre^GCS^f/f^* and WT mice as measured by IFNγ secretion (Figure 7D).

In line with the latter result, the general T cell population of *Vav^Cre^GCS^f/f^* mice was unaffected and showed an unaltered production of IFNγ in response to TCR-independent (PMA/calcium ionophore) or TCR-dependent (CD3/CD28) stimulation (Figure 7E).

## Discussion

Although substantial progress in understanding the function of iNKT cells has been achieved since their discovery two decades ago, the identity of the endogenous lipid antigen(s) mediating their thymic positive selection and peripheral activation remains largely elusive. Originally, it has been shown that cells deficient in GlcCer-based GSL were unable to stimulate iNKT cell hybridomas, thus implicating that the endogenous ligand might be GlcCer or a GlcCer-derived GSL (Figure 1) (44). However, mice deficient for singular series of GlcCer-derived GSL such as gangliosides, globosides, isoglobosides, and sulfatides were shown to have normal iNKT cell numbers; thus, casting doubts upon a decisive role of GlcCer-derived GSL in the positive selection of iNKT cells [(53, 56) and own unpublished results]. Similarly, βGalCer-derived GSL were demonstrated to be dispensable for iNKT cell development (44). Interestingly, mice deficient in several GSL-degrading enzymes (α-galactosidase A, β-galactosidase, β-hexosaminidase A/B, Niemann-Pick-disease type C1-protein) have significantly reduced iNKT cell numbers (64). In case of α-galactosidase A-deficient mice, which store globosides and isoglobosides, Darmoise et al. attributed the diminished iNKT cell population to excessive levels of the isogloboside iGb3 that would elicit apoptosis of iNKT cells by continuous overstimulation (65). However, using a genetic approach, we could show that in α-galactosidase A-deficient mice, the reduction of iNKT cells was a consequence of lysosomal dysfunction and not of iGb3 *per se* (56).

The paradox that iNKT cell development remains unaltered after depletion of singular GlcCer-derived GSL groups offers three explanations: (a) lipids other than GSL, (b) other—yet unaddressed—GlcCer-derived GSL, or (c) the GlcCer itself mediate the positive selection of iNKT cells. Several lines of evidence have shown that also lipids other than GSL might be important for the iNKT cell activation and development (38, 43). The first publication has demonstrated that ether-bonded mono-alkyl glycerophosphates stimulated iNKT cells and that deficiency for GNPAT led to an approximately 50% reduction of the iNKT cell population *in vivo* (43). However, GNPAT-deficient mice have multiple severe abnormalities and those that survive develop hypomorphism (66), altogether making the exclusion of any unspecific effects on the iNKT cell population very challenging. By contrast, Brennan et al. have pinpointed to βGlcCer as the self-antigen responsible for activation of iNKT cells by dendritic cells upon recognition of microbial danger signals (45). However, their later findings implicated that not βGlcCer but a rare, yet unknown, component of the GlcCer fraction should be responsible for the stimulation of iNKT cells (46). Recently, Kain et al. could identify trace amounts of α-anomeric GSL in mammalian immune cells and demonstrate their stimulatory capacity toward iNKT cells (67). Independently of these ambiguous results on GlcCer with regard to its stimulatory role for iNKT cells in the periphery, it remained unknown whether GlcCer-derived GSL (be it α- or β-anomers) might represent also the endogenous lipid antigen in the process of thymic iNKT cell selection.

We describe here the first functional *in vivo* model that has allowed for a depletion of GlcCer and GlcCer-derived GSL in DP thymocytes and that has shown a significant reduction in iNKT cells. Due to the very early activation of the Vav-cre promoter in hematopoietic progenitors (51), it was possible to achieve not only a deletion of the GCS mRNA but also a highly efficient depletion of its product GlcCer that averaged at 99.6% in DP thymocytes. The finding of the residual 0.4% GlcCer in *Vav^Cre^GCS^f/f^* DP thymocytes might have several explanations: (a) it represents residual, not yet catabolized, traces of intrinsic GlcCer in *Vav^Cre^GCS^f/f^* DP thymocytes. (b) Thymocytes could potentially utilize blood-derived GSL *in vivo* as it has been shown also for other cell types (68). Of note, we have omitted any exposure of the thymocytes to fetal calf serum or albumin during their *ex vivo* preparation and sorting. (c) Contamination by epithelial cells or cell fragments before or during the sorting might have artificially contributed to the measured residual GlcCer levels. For conventional T cells, it has been demonstrated that even a single antigen–MHC complex can elicit their activation (69, 70). Therefore, it cannot be excluded that also such trace amounts of GlcCer still found on DP thymocytes would enable sufficient activation of iNKT cells and thus be—at least partially—responsible for the remaining approximately 50% of iNKT cells in *Vav^Cre^GCS^f/f^* mice. Alternatively, the coexistence of multiple endogenous antigens might explain the incomplete reduction of iNKT cells upon 99.6% reduction of GlcCer. The aforementioned work by Facciotti et al. describing also an approximately 50% reduction of iNKT cells in GNPAT-deficient mice might indeed support such an assumption and speak in favor of a coexistence of GlcCer and GNPAT-derived ligands. In addition, it has been demonstrated that iNKT cells recognize also other self-lipids that can be loaded on CD1 molecules (e.g., phosphatidylinositol, phosphatidylethanolamine, lysophospholipids, sphingolipids) (36–39, 71, 72). The *in vivo* role of these lipids and their possibly interchangeable function will have to be addressed by further studies.

In view of the potential ligand heterogeneity, it might be speculated that the depletion of one ligand might alter the CD1d–TCR interaction and indirectly influence the Vβ repertoire. However, the TCR Vβ repertoire remained unchanged in *Vav^Cre^GCS^f/f^* mice. In iNKT cells, the interaction between CD1d-bound antigen and the TCR is mediated mainly by the TCR α-chain that is also in contact with the antigen. The β-chain, as contrasted to the conventional T cells, contacts the CD1d molecule only marginally with a minimal, if any, access to the lipid antigen (17, 18). This might offer an explanation of the fact that the antigen depletion did not elicit any alteration of the TCRVβ repertoire in *Vav^Cre^GCS^f/f^* mice.

The finding of unaltered conventional T cells in *Vav^Cre^GCS^f/f^* mice is surprising because GSL contribute to the formation of membrane microdomains that are important for the signal transduction. However, our results indicate that GlcCer-derived GSL are dispensable for the conventional T cell population as their thymic development, peripheral frequency and response to TCR-dependent and -independent stimulation were unaffected in *Vav^Cre^GCS^f/f^* mice. In line with this, *in vitro* and *in vivo* activation of iNKT cells by the exogenous antigen αGalCer was unaffected in *Vav^Cre^GCS^f/f^* mice. Thus, it seems unlikely that the reduction of iNKT cells would be a consequence of an unspecific or cell-intrinsic T cell phenotype.

The presented results also have shown that expression of SLAM, Ly108, and CD1d on DP thymocytes does not depend on GlcCer and GlcCer-derived GSL. The described shift of CD1d from late to early endosomes remains as yet unexplained. However, the normal expression of CD1d in lysosomes and on the cell surface together with the unaltered antigen presentation on *Vav^Cre^GCS^f/f^* DP thymocytes make a functional impact of GlcCer-derived GSL on the processes of antigen presentation unlikely. This corresponds to previous *in vitro* observations that CD1d expression levels and presentation of exogenous synthetic antigens were unaffected in GSL-deficient cells (38). In view of the unaltered CD1d functionality and normal expression of SLAM and Ly108 on DP thymocytes, the reduced proliferation and increased apoptosis of *Vav^Cre^GCS^f/f^* iNKT cells (but not conventional thymocytes) speaks in favor of a diminished presentation of an endogenous antigen on DP thymocytes.

We have analyzed the GSL spectra of WT DP thymocytes and found that they expressed a hexosylceramide compound that was absent in thymocytes deficient for GCS, thus implicating that it was GlcCer and not GalCer. In addition, DP thymocytes expressed also gangliosides such as GM1a, GM1b, GD1b, and GD1c. These results agree with a recent report analyzing the GSL composition of unsorted thymocytes and CD4- and CD8-positive T cells (73). However, gangliosides unlikely represent the iNKT selecting endogenous ligands as mice deficient for ganglioside-synthesizing enzymes were shown to have normal iNKT cell populations (56, 74). These findings pinpoint to GlcCer (and not its downstream metabolites gangliosides) as a GSL present on DP thymocytes and of importance in iNKT cell selection.

In summary, our results demonstrate *in vivo* that GCS-dependent GSL, in particular GlcCer, influence the homeostatic iNKT cell development.

## Ethics Statement

Animal experiments were performed in compliance with the German guidelines on animal protection and approved by the committee (Regierungspräsidium Karlsruhe).

## Author Contributions

SP initiated the study and wrote the manuscript. ZP, MR, RJ, DK, RS, and SP performed and evaluated experiments. H-JG provided animals, reagents, and technical assistance and gave critical input to the study and to the manuscript.

## Conflict of Interest Statement

The authors declare that the research was conducted in the absence of any commercial or financial relationships that could be construed as a potential conflict of interest.
