# Supplementary material for: Glucosylceramide Synthase Is Involved in Development of Invariant Natural Killer T Cells
Source: Front Immunol. 2017 Jul 21;8:848. doi: 10.3389/fimmu.2017.00848 (PMC5519558; doi:10.3389/fimmu.2017.00848)
Supplement: Supplementary file 1 [file table_1.doc]

Supplementary Material

Glucosylceramide synthase is involved in development of invariant natural killer T cells.

**Zoran V. Popovic^*^, Mariona Rabionet, Richard Jennemann, Damir Krunic, Roger Sandhoff, Hermann-Josef Gröne and Stefan Porubsky^*^**

*** Correspondence:** Corresponding Author: z.popovic@dkfz.de

# Supplementary Table 1: Comparison of relative and absolute iNKT cell numbers between *Vav^Cre^* -positive (*Vav^Cre^GCS^+/+^*) and *Vav^Cre^* -negative (*Vav^WT^GCS^+/+^*) mice.

|  | *Vav^Cre^GCS^+/+^* | *Vav^WT^GCS^+/+^* | P (t-test) |
| --- | --- | --- | --- |
| thymus, relative (%) | 0.38 ± 0.02 | 0.39 ± 0.03 | ns |
| thymus, absolute (10^6^ cells) | 0.81 ± 0.03 | 0.95 ± 0.28 | ns |
| spleen, relative (%) | 0.98 ± 0.04 | 1.49 ± 0.72 | ns |
| spleen, absolute (10^6^ cells) | 0.37 ± 0.07 | 0.44 ± 0.21 | ns |
| liver, relative (%) | 21.43 ± 3.29 | 27.30 ± 3.12 | ns |
| liver, absolute (10^6^ cells) | 6.63 ± 1.99 | 4.91 ± 0.36 | ns |

Shown are means ± SEM; N = 3/group; ns, non significant.
